# Polar Vantage and Oura Physical Activity and Sleep Trackers: Validation and Comparison Study

**DOI:** 10.2196/27248

**Published:** 2022-05-27

**Authors:** André Henriksen, Frode Svartdal, Sameline Grimsgaard, Gunnar Hartvigsen, Laila Arnesdatter Hopstock

**Affiliations:** 1 Department of Computer Science UiT The Arctic University of Norway Troms Norway; 2 Department of Psychology UiT The Arctic University of Norway Tromsø Norway; 3 Department of Community Medicine UiT The Arctic University of Norway Tromsø Norway; 4 Department of Health and Nursing Sciences University of Agder Grimstad Norway

**Keywords:** actigraphy, fitness trackers, motor activity, energy expenditure, steps, activity tracker

## Abstract

**Background:**

Consumer-based activity trackers are increasingly used in research, as they have the potential to promote increased physical activity and can be used for estimating physical activity among participants. However, the accuracy of newer consumer-based devices is mostly unknown, and validation studies are needed.

**Objective:**

The objective of this study was to compare the Polar Vantage watch (Polar Electro Oy) and Oura ring (generation 2; Ōura Health Oy) activity trackers to research-based instruments for measuring physical activity, total energy expenditure, resting heart rate, and sleep duration in free-living adults.

**Methods:**

A total of 21 participants wore 2 consumer-based activity trackers (Polar watch and Oura ring), an ActiGraph accelerometer (ActiGraph LLC), and an Actiheart accelerometer and heart rate monitor (CamNtech Ltd) and completed a sleep diary for up to 7 days. We assessed Polar watch and Oura ring validity and comparability for measuring physical activity, total energy expenditure, resting heart rate (Oura), and sleep duration. We analyzed repeated measures correlations, Bland-Altman plots, and mean absolute percentage errors.

**Results:**

The Polar watch and Oura ring values strongly correlated (*P*<.001) with the ActiGraph values for steps (Polar: *r*=0.75, 95% CI 0.54-0.92; Oura: *r*=0.77, 95% CI 0.62-0.87), moderate-to-vigorous physical activity (Polar: *r*=0.76, 95% CI 0.62-0.88; Oura: *r*=0.70, 95% CI 0.49-0.82), and total energy expenditure (Polar: *r*=0.69, 95% CI 0.48-0.88; Oura: *r*=0.70, 95% CI 0.51-0.83) and strongly or very strongly correlated (*P*<.001) with the sleep diary–derived sleep durations (Polar: *r*=0.74, 95% CI 0.56-0.88; Oura: *r*=0.82, 95% CI 0.68-0.91). Oura ring–derived resting heart rates had a very strong correlation (*P*<.001) with the Actiheart-derived resting heart rates (*r*=0.9, 95% CI 0.85-0.96). However, the mean absolute percentage error was high for all variables except Oura ring–derived sleep duration (10%) and resting heart rate (3%), which the Oura ring underreported on average by 1 beat per minute.

**Conclusions:**

The Oura ring can potentially be used as an alternative to the Actiheart to measure resting heart rate. As for sleep duration, the Polar watch and Oura ring can potentially be used as replacements for a manual sleep diary, depending on the acceptable error. Neither the Polar watch nor the Oura ring can replace the ActiGraph when it comes to measuring steps, moderate-to-vigorous physical activity, and total energy expenditure, but they may be used as additional sources of physical activity measures in some settings. On average, the Polar Vantage watch reported higher outputs compared to those reported by the Oura ring for steps, moderate-to-vigorous physical activity, and total energy expenditure.

## Introduction

In a research setting, accelerometers are often used to objectively measure movement behavior (eg, sleep, physical activity, and sedentary behavior). Device outputs are converted into various estimates for physical activity, energy expenditure, sleep, and heart rate (for devices with a heart rate sensor). A wide range of devices exist for both research [1] and the consumer market [2].

Consumer-based activity trackers are increasingly used in research, as they have the potential to increase activity participation [3] and can be used for estimating physical activity and related variables [2,4,5]. Compared to research-based accelerometers and heart rate sensors, consumer-based activity trackers are often cheaper and less intrusive and have increased battery and storage capacity. They are also associated with less participant burden than that associated with self-report instruments, such as physical activity and sleep diaries. Sleep diaries [6] are less resource demanding than polysomnography, but compliance can be challenging [7]. Consumer-based activity trackers that measure sleep duration can therefore be a potential replacement for sleep diaries.

The accuracy of newer consumer-based devices is mostly unknown, and validation studies are needed. The validity of consumer-based activity trackers can be studied via comparisons against research-based accelerometers (eg, ActiGraph [ActiGraph LLC]) that, in turn, have been validated against gold standard methods. The Polar Vantage watch (Polar Electro Oy) and the Oura ring (Ōura Health Oy) are 2 new consumer-based activity trackers that can potentially replace, or be used in addition to, existing research-based accelerometers or self-report tools. However, before using these trackers, there is a need to test their accuracy.

A previous lab-based validation study of Polar Vantage devices found that the devices estimated energy expenditure within 20% of the actual value (commonly deemed “acceptable” in the literature) in 59.5% of all cases; however, this error rate varied depending on the activity type (ranging from 27% to 93%) [8]. No validation or comparison study on physical activity or sleep has been conducted on the Polar Vantage watch to date, and no study on this activity tracker has been done with free-living populations. Similarly, de Zambotti et al [9] compared the Oura ring to polysomnography and found that although further validation studies are needed, the Oura ring has the potential to be used as a tool for detecting the time spent in different sleep phases in studies conducted with free-living populations. However, there are no previous validation studies that have tested the validity of the physical activity or energy expenditure measures of the Oura ring.

The aim of this study on free-living adults was therefore to test the validity and comparability of the physical activity and energy expenditure measures of the Polar Vantage watch and Oura ring by comparing them to those of the ActiGraph. We also compared the resting heart rates (RHRs) measured by the Oura ring to those measured by Actiheart electrocardiograms (CamNtech Ltd). In addition, we compared sleep durations from a sleep diary and outputs from the Polar Vantage watch and Oura ring.

## Methods

### Instruments

The Polar Vantage activity tracker (Polar Electro Oy) was released in 2018, and it is equipped with a 50-Hz (ie, 50 measurements per second) triaxial accelerometer for physical activity tracking. It weighs 45 to 66 g, has 1 week of battery life, and comes in multiple strap and metal casing colors. The Polar Vantage watch is a multisport watch that is to be worn on the wrist.

We used Polar Flow (Polar Electro Oy) to download daily Polar Vantage variables for steps, moderate-to-vigorous physical activity (MVPA), total energy expenditure (TEE), and sleep duration (sleep time).

The Oura activity and sleep ring (Ōura Health Oy) was released in 2018, and it is equipped with a 50-Hz triaxial accelerometer for physical activity tracking and a photoplethysmograph with 2 infrared light-emitting diodes for optical pulse measurements. It comes in sizes US 6 to US 13, weighs 4 to 6 g, has 6 days of battery life, and comes in different shapes and colors. The Oura ring is a smart ring that is to be worn on the finger and focuses on sleep and well-being by combining physical activity and heart rate parameters.

The Oura Generation 2 cloud dashboard was used to download daily Oura variables for steps, MVPA, TEE, sleep duration (total sleep), and RHR.

The ActiGraph wGT3X-BT (ActiGraph LLC) is a triaxial accelerometer. The sample rate can be set to 30 to 100 Hz. It weighs 19 g and has up to 25 days of battery life. It is extensively used to estimate activity in free-living research, as it provides reasonable estimates for physical activity intensity [10], steps [11], and energy expenditure [12].

We used ActiLife (ActiGraph LLC) to download ActiGraph accelerometer data and generate variables. We used triaxial activity (ie, vector magnitude [VM]) counts to generate physical activity and energy expenditure variables in addition to step counts, which were reported directly. We calculated minutes of MVPA by using the cut points defined by Sasaki et al [10] (ie, >2690 VMs). Activity energy expenditure was calculated by using the “Freedson VM3 combination 2011 + Williams work-energy equation” [10] and was converted to TEE by using the Schofield equation [13] for resting energy expenditure and subtracting 10% of the original energy expenditure value to account for diet-induced thermogenesis. Although the ActiGraph is an objective instrument, researchers must make subjective choices (eg, cut points, sample rate, and wear location) that can influence output variables and thus affect conclusions.

The Actiheart 4 (CamNtech Ltd) records heart rate by using a 1-lead electrocardiogram with a 128-Hz sampling rate. It weighs less than 10 g and is attached to the chest by using 2 standard electrocardiogram electrodes. Actiheart is valid and reliable for heart rate detection [14].

The Actiheart software was used to download sleeping (ie, resting) heart rate data from the Actiheart (CamNtech Ltd). Sleep duration (time in bed) was calculated based on manually recorded times (ie, time getting out of bed minus time getting into bed) from the sleep diary. We did not analyze sleep duration by using the Actiheart software, as this feature is considered experimental.

We only included valid days in the analysis, which were defined as days with at least 10 hours of wear time [15]. Wear time for the ActiGraph was analyzed by using the ActiLife default settings for the Troiano [16] wear time algorithm. The wear time algorithms for Polar Vantage and Oura are unknown.

The sleep diary contained a subset of questions from the Consensus Sleep Diary [17] that were relevant to sleep duration, which was measured as the time in bed. Specifically, it contained question 1 (“What time did you get into bed?”) and question 7 (“What time did you get out of bed for the day?”).

### Participants and Procedure

We used convenience sampling to recruit 21 participants. Participants were eligible for inclusion if they were above 18 years of age, had normal physical function, and were willing to wear all 4 devices and keep a sleep diary for 5 days. Participants were recruited among university students and staff and people who were close to these participants.

**Figure 1:**
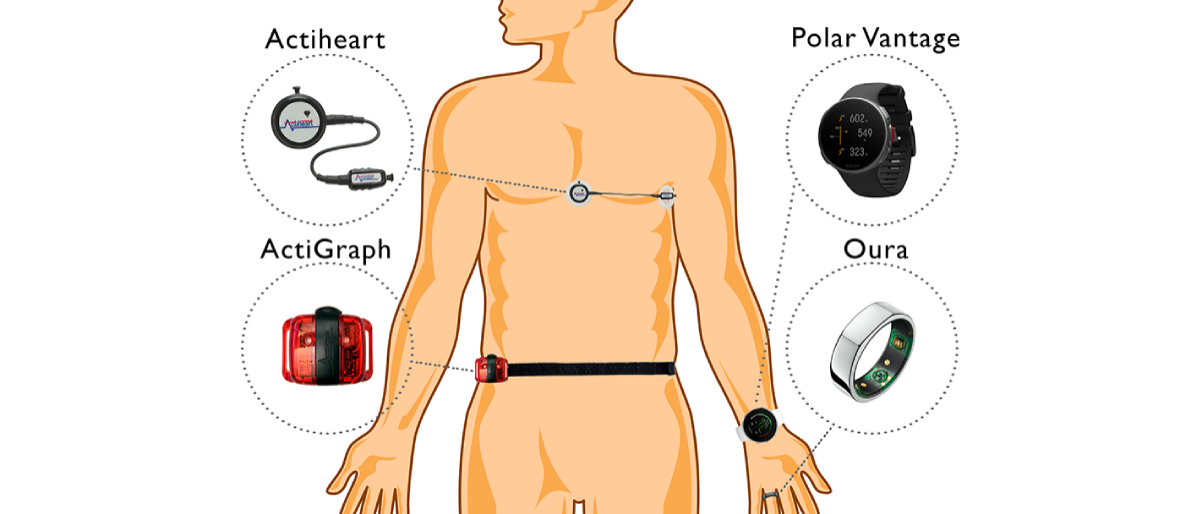
Illustration of instrument placements for the Actiheart, Polar Vantage watch, ActiGraph, and Oura ring.

We initialized devices with self-reported information on height, weight, age, sex, and dominant hand. Participants wore the Polar Vantage watch and Oura ring on their nondominant hand. The ActiGraph was set up for 100-Hz recording and placed on the right hip (attached via an elastic band). The Actiheart was placed at the level of the fifth intercostal space on the sternum (medial part) and to the left (lateral part) and was attached via two 3M Red Dot 2238 electrodes (3M Company). Participants were asked to wear all 4 devices simultaneously and complete the sleep diary for 5 days. They were instructed to complete the sleep diary (time getting into bed and time getting out of bed) upon waking every morning. Since the devices were placed on participants on day 1 and removed on day 5, the expected number of valid days per person was up to 3. Data were collected from May to June 2019. Figure 1 shows the device placements.

An overview of instrument details and the software used for instrument setup, data download, and variable generation is given in Table 1.

**Table 1:** Overview of the instrument suppliers, models, firmware versions, and software versions used to set up instruments, download data, and generate output variables.

Characteristic	Instrument			
	Actiheart	Polar Vantage watch	ActiGraph	Oura ring
Supplier	CamNTech	Polar Electro	ActiGraph	Oura
Model	4	V/M	wGT3X-BT	2P
Firmware version	H90.65	3.2.10	1.9.2	1.91.1
Software for instrument setup, data download, and variable generation	Actiheart 5.1.10	Polar Flow [18]	ActiLife 6.13.3	Oura app [19]

### Statistics

The ActiGraph was used as a reference monitor for steps, MVPA, and TEE. The Actiheart was used as a criterion measure for RHR. The sleep diary (time in bed) was used to compare sleep durations (Polar: sleep time; Oura: total sleep). Normality was tested by using the Shapiro-Wilkins test, and bootstrapping was used on all variables. Correlations were calculated by using repeated measures correlations [20-22] with the correlation cutoffs suggested by Evans [23] (ie, very weak: <0.2; weak: 0.2-0.4; moderate: 0.4-0.6; strong: 0.6-0.8; very strong: >0.8). Mean absolute percentage error (MAPE) was calculated across person-day cases to assess measurement errors between each instrument and the reference monitor. Although there is no universally accepted threshold for MAPE, a common practice that was established in prior validation studies conducted with free-living populations is to use 10% as a cutoff to indicate low error [24,25]. We therefore used a below-10% cutoff to indicate low or acceptable error and an above-10% cutoff to indicate high error. Some participants had a high level of MVPA based on the Polar Vantage watch but a low level of MVPA based on the ActiGraph. We conducted a subanalysis in which we excluded these outlier participants and recalculated MAPEs.

Bland-Altman plots for multiple measurements were created to determine the agreement between each instrument and the reference monitor, as well as the agreement between instruments (Polar watch and Oura ring) [26]. This is, according to Bland and Altman [26], an appropriate approach for assessing the agreement between methods when there is an inequal number of observations per participant.

### Ethics Approval

The Norwegian Regional Committees for Medical and Health Research Ethics North reviewed this study (approval number: 2019/557/REK nord). Participants received written and oral instructions and gave informed consent. This study was conducted in accordance with the 1964 Declaration of Helsinki and its later amendments.

## Results

### Participant Characteristics

Summary statistics for participants’ age, sex, height, weight, and BMI, are given in Table 2.

Each participant had 2 to 6 valid days of physical activity recorded, totaling 57 and 68 valid person-days of simultaneous ActiGraph and activity tracker usage for the Polar Vantage watch and Oura ring, respectively. On average, each participant simultaneously wore the ActiGraph and Polar Vantage watch for 2.7 days and the ActiGraph and Oura ring for 3.2 days. Participants manually recorded 0 to 5 days of sleep, totaling 48 and 44 person-days of sleep diary and activity tracker recordings for the Polar Vantage watch and Oura ring, respectively. On average each participant kept a sleep diary for 2.3 days. There were 39 person-days of Oura and Actiheart RHR recordings, averaging 1.8 person-days of recordings per participant.

**Table 2:** Participants’ age, sex, height, weight, and BMI (N=21).

Variable	Value
Age (years), mean (SD; range)	33 (14; 22-71)
Men, n (%)	57 (12)
Height (cm), mean (SD; range)	176 (9; 160-190)
Weight (kg), mean (SD; range)	79 (13; 57-103)
BMI (kg/m^2^), mean (SD; range)	26 (4; 18-35)

### Correlation, Error, and Agreement

Table 3 presents valid person-days, correlations, MAPEs, and mean differences (with limits of agreement) for the step, MVPA, TEE, sleep duration, and RHR (Oura) measures of the Polar Vantage watch and the Oura ring, which were compared to the criterion measure. Table 4 presents the same variables (except RHR), which were used to compare the Polar Vantage watch with the Oura ring directly.

**Figure 2:**
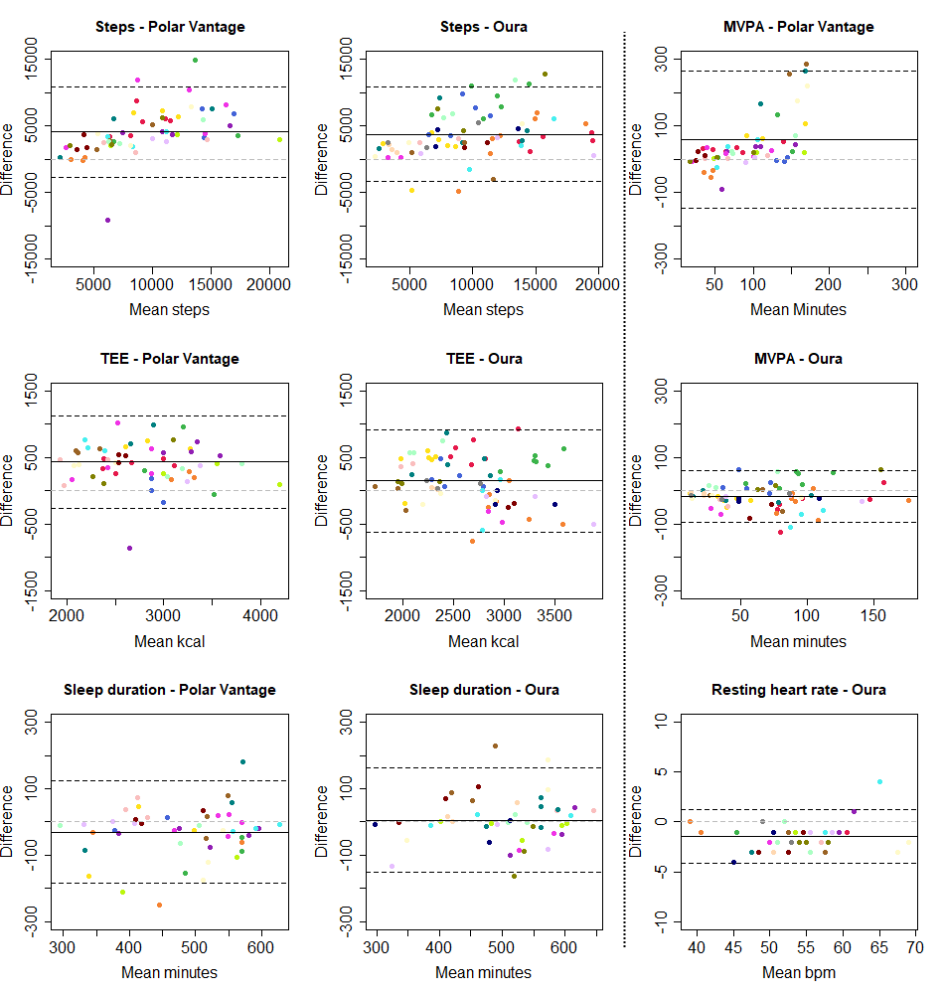
Bland-Altman plots for comparing the Polar Vantage watch and Oura ring to the ActiGraph. Participants are represented by different colors. Black solid lines represent the mean difference, black dashed lines represent the limits of agreement, and grey dashed lines represent the line of equivalence. bpm: beats per minute; MVPA: moderate-to-vigorous physical activity; TEE: total energy expenditure.

**Figure 3:**
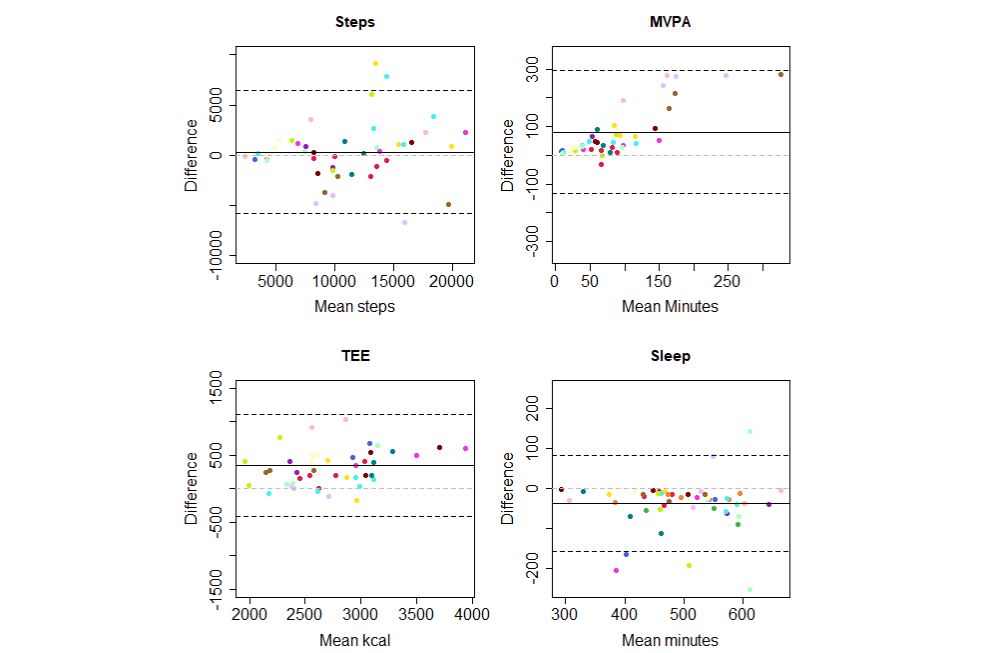
Bland-Altman plots for comparing the Polar Vantage watch and Oura ring measures for steps, moderate-to-vigorous physical activity (MVPA), total energy expenditure (TEE), and sleep duration. Participants are represented by different colors. Black solid lines represent the mean difference, black dashed lines represent the limits of agreement, and grey dashed lines represent the line of equivalence.

The mean differences are illustrated in Figure 2 by using Bland-Altman plots. Each participant is represented by a different color. Similarly, Figure 3 further illustrates how the Polar Vantage watch directly compares to the Oura ring by using Bland-Altman plots. In Figure 3, a mean difference above the line of equivalence indicates that the Polar Vantage watch reports higher numbers on average compared to those reported by the Oura ring.

Step counts were strongly correlated between the reference monitor and both the Polar Vantage watch and the Oura ring. Bland-Altman plots showed that both the Polar Vantage watch and the Oura ring overreported steps. The MAPE was high for both activity trackers. The correlation between the Polar Vantage watch and Oura ring was very strong for steps. On average, the Polar Vantage watch reported 305 additional steps per day when compared to those reported by the Oura ring.

MVPA values were strongly correlated between the reference monitor and both activity trackers. The Polar Vantage watch overreported MVPA values, while the Oura ring underreported MVPA values. For the Polar Vantage watch, overreporting was higher for higher values of MVPA. The limit of agreement ranges in the Bland-Altman plots were also wider for the Polar Vantage watch. MAPEs were high, but the Oura ring had lower mean errors compared to those of the Polar Vantage watch. We identified 3 participants for whom the Polar Vantage watch reported a high MVPA value and the ActiGraph reported a low MVPA value. The MAPE subanalysis, in which these three participants were excluded, showed a MAPE of 49%—a decrease from 143%. The correlation between the Polar Vantage watch and Oura ring was very strong for MVPA. On average the Polar Vantage watch reported 81 additional minutes of MVPA per day when compared to those reported by the Oura ring.

TEE values were strongly correlated between the reference monitor and both activity trackers. Both activity trackers overreported TEE, but the Polar Vantage watch overreported TEE at a higher rate. Both MAPEs were higher than the 10% cutoff for acceptable error. The correlation between the Polar Vantage watch and Oura ring was very strong for TEE. On average, the Polar Vantage watch reported 349 additional kcal per day when compared to those reported by the Oura ring.

Sleep durations were strongly correlated between the diary and the Polar Vantage watch and were underreported by a mean of 30 minutes by the Polar Vantage. The Oura ring–derived sleep durations very strongly correlated with the diary-derived sleep durations but were overreported on average by 6 minutes. Both activity trackers had a borderline acceptable MAPE. The correlation between the Polar Vantage watch and Oura ring was strong for sleep duration. On average, the Polar Vantage watch reported 38 fewer minutes of sleep per day when compared to those reported by the Oura ring.

RHRs were very strongly correlated between the criterion measure and the Oura ring. The MAPE was low (3%), and on average, the Oura ring underreported RHR by 1 beat per minute.

**Table 3:** Repeated measures correlation (RMC), mean absolute percentage error (MAPE), and mean difference for each Polar Vantage watch and Oura ring variable.

Measure		Person-days, n	RMC^a^ (95% CI)	MAPE	Mean difference (lower LoA^b^, upper LoA)
**Polar Vantage watch**					
	Steps	57	0.75 (0.54-0.92)	72	4091 (−2693, 10 876)
	MVPA^c^	57	0.76 (0.62-0.88)	143	59 (−148, 266)
	Total energy expenditure	57	0.69 (0.48-0.88)	19	430 (−267, 1127)
	Sleep duration	48	0.74 (0.56-0.88)	11	−30 (−183, 123)
**Oura Generation 2 ring**					
	Steps	68	0.77 (0.62-0.87)	69	3779 (−3361, 10 919)
	MVPA	68	0.70 (0.49-0.82)	49	−18 (−96, 61)
	Total energy expenditure	68	0.70 (0.51-0.83)	13	148 (−624, 920)
	Sleep duration	44	0.82 (0.68-0.91)	11	6 (−152, 164)
	Resting heart rate	39	0.90 (0.85-0.96)	3	−1 (−4, 1)

^a^All RMC *P* values are <.001.

^b^LoA: limit of agreement.

^c^MVPA: moderate-to-vigorous physical activity.

**Table 4:** Repeated measures correlations (RMCs) and mean differences between Polar Vantage watch and Oura watch variables.

Measure	Person-days, n	RMC^a^ (95% CI)	Mean difference (lower LoA^b^, upper LoA)
Steps	44	0.89 (0.77-0.97)	305 (−5832, 6441)
MVPA^c^	44	0.82 (0.69-0.91)	81 (−133, 296)
Total energy expenditure	44	0.85 (0.75-0.93)	349 (−411, 1109)
Sleep duration	51	0.74 (0.49-0.93)	−38 (−157, 82)

^a^All RMC *P* values are <.001.

^b^LoA: limit of agreement.

^c^MVPA: moderate-to-vigorous physical activity.

## Discussion

### Principal Findings

Step, MVPA, and TEE outputs from the activity trackers all strongly correlated with those of the ActiGraph. The MAPEs were high for these variables, with lower errors recorded for the Oura ring compared to those recorded for the Polar Vantage watch. Sleep durations were strongly to very strongly correlated between the sleep diary and both the Polar Vantage watch and the Oura ring. In addition, RHRs recorded by the Oura ring very strongly correlated with those recorded by the Actiheart. The MAPEs for the sleep diary were borderline acceptable, while the MAPEs for RHR were acceptable. The correlations between the Polar Vantage watch and Oura ring were very strong for steps, MVPA, and TEE and were strong for sleep duration.

The CI range was wide for all correlations and was borderline acceptable for Oura ring–derived RHR. The wide ranges are not surprising, given the low sample size. The MAPEs were very high for some comparisons, especially for MVPA and the Polar Vantage watch. In the MAPE subanalysis for the Polar Vantage watch and the ActiGraph, the MAPE dropped from 143% to 49%. This is well above the 10% threshold.

### Strength and Limitations

The major strength of this study is the inclusion of multiple days of recordings for each participant instead of 1 mean measure per participant. Using multiple measurements per participant increases the width of the limits of agreement and thus provides a more accurate representation of the actual agreement between methods [26]. A further strength is the gender balance and the wide range of ages, heights, weights, and BMIs among the participants. This is also the first study to assess the accuracy of physical activity estimates from the Oura ring. The major weakness of this study is the use of non–gold standard methods for comparing step, MVPA, and energy expenditure values. However, there is no gold-standard for estimating physical activity in free-living populations. The ActiGraph has been previously validated using gold standard methods [10-12] and is an appropriate reference monitor for studies conducted in free-living settings. The gold standards for estimating energy expenditure and sleep duration are the doubly labeled method and polysomnography, respectively. We did not have access to these two methods.

### Comparison With Prior Work

Although the correlation for Polar Vantage watch–derived energy expenditure in this study (*r*=0.69) is stronger than those in most previous studies on Polar Vantage devices (only discontinued devices) [27], it is weaker compared to those in a 2019 lab-setting study on a Polar Vantage device (*r*=0.89) [8] and a 2018 free-living study on the Polar M430 (*r*=0.91) [28]. The correlations for MVPA values in previous Polar device validation studies vary, but our findings (*r*=0.76) are as strong as the findings from a study on the Polar M430 (*r*=0.75) [28]. Similarly, the strong correlation (*r*=0.75) for step counts is in accordance with those in previous studies on the Polar M430 (*r*=0.85) [28] and the Polar V800 (*r*=0.89-0.92) [29].

For the Oura ring, we could not find any previous studies on steps, MVPA, or TEE. Even though the correlations were strong in this study, most results showed that the measurement error was high, and most variables were overreported when compared to those reported by the reference monitor. Thus, we cannot recommend replacing existing research-based accelerometers with the activity trackers investigated in this study. However, as additional instruments for long-term physical activity recording, these consumer-based activity trackers can potentially provide additional value to studies examining changes in physical activity over time. TEE may be especially interesting to measure over time, since it had close to acceptable errors (range: 13%-19% mean error).

We could not find any previous validation studies on sleep duration for the Polar Vantage watch. However, the strong correlation (*r*=0.74) in this study is in accordance with the findings for an earlier Polar watch model (Polar A370); its sensitivity for correctly detecting sleep duration was >0.91 [4]. Sleep duration was underreported in both studies, which is expected since sleep onset and sleep offset are generally greater than 0 minutes. The Oura ring overreported sleep duration but only by an average of 6 minutes per night. This is in accordance with the findings of de Zambotti et al [9], who concluded that the Oura ring shows “promising results” for sleep detection (ie, a 96% sensitivity for detecting sleep) in their study involving polysomnography. Both the Polar Vantage watch and Oura ring can provide reasonably close estimates for sleep duration with a 10% to 13% average error, which is close to the acceptable cutoff. This is especially interesting for long-term sleep monitoring, as keeping a manual sleep diary is prone to low compliance [7], and objective wrist-worn devices can provide more accurate results [30].

The strong correlation and low average error found for the Oura ring–derived RHR is in accordance with heart rate measurements from some wrist-worn activity trackers [5,31]. A recent validation study on Oura ring–derived sleeping heart rate also reported high agreement (Spearman correlation: 0.996) between the Oura ring and an electrocardiogram [32]. Compared to the Actiheart and other similar research instruments, the Oura ring is a low-burden device that is capable of collecting various heart rate measures over several months. This makes it an interesting instrument for future research, when long-term heart rate measuring is of interest.

Several systematic reviews on consumer-based activity tracker validity have been published in the last few years, and they reported similar conclusions [27,31,33-38]. Fuller et al [31] published a large systematic review in 2020 that assessed the validity of step, energy expenditure, and heart rate estimates for devices from Apple, Fitbit, Garmin, Mio, Misfit, Polar, Samsung, Withings, and Xiaomi. Based on the 148 included validation studies, they concluded that although there is variation, consumer-based activity trackers can accurately measure steps and heart rates in lab settings. The most recent systematic review on Garmin activity trackers by Evenson et al [38] reported high validity for step counts (good to excellent correlation coefficients and acceptable MAPEs) but low validity (large variations in correlation coefficients and high MAPEs) for energy expenditure values and heart rates. A systematic review and meta-analysis on energy expenditure by O’Driscoll et al [37] further showed that accuracy is dependent on the type of activity performed and that trackers with heart rate monitors have lower measurement errors. The Polar Vantage watch reported on average higher numbers compared to those reported by the Oura ring.

Although several systematic reviews have been published [27,31,37,38], these reviewed activity trackers that are now discontinued. This highlights the need for continuous validation studies on new devices, as suggested by Fuller et al [31].

### Conclusions

The Oura ring can be used as an alternative to the Actiheart to measure RHR unless very high accuracy is required. Similarly, the Polar Vantage watch and Oura ring can potentially be used as replacements for a manual sleep diary for sleep duration measurements. Neither the Polar Vantage watch nor the Oura ring can replace the ActiGraph when it comes to measuring steps, MVPA, and TEE, but they may be used as additional sources of physical activity measures in some settings.

## Abbreviations

MAPE: mean absolute percentage error
MVPA: moderate-to-vigorous physical activity
RHR: resting heart rate
TEE: total energy expenditure
VM: vector magnitude
